# The associations of daily steps and body mass index with incident gastroesophageal reflux disease in older adults

**DOI:** 10.3389/fspor.2024.1384845

**Published:** 2024-04-05

**Authors:** Joey M. Saavedra, Elizabeth C. Lefferts, Bong Kil Song, Duck-chul Lee

**Affiliations:** ^1^ Department of Kinesiology, Iowa State University, Ames, IA, United States; ^2^ Department of Physical Education, Seoul National University, Seoul, Republic of Korea

**Keywords:** daily steps, body mass index, gastroesophageal reflux disease, older adults, epidemiology

## Abstract

**Background:**

High body mass index (BMI) is a major risk factor of gastroesophageal reflux disease (GERD), a prevalent morbidity of older adulthood linked to lower quality of life and an increased risk of esophageal cancers. Daily stepping behavior, the most common physical activity of older adulthood, is associated with an array of favorable health outcomes, sometimes independent of high BMI. Whether stepping behavior is associated with the incidence of GERD independently or in combination with BMI is currently unclear.

**Materials and methods:**

We followed 442 individuals (58.4% female) aged 65–91 years enrolled in the Physical Activity and Aging Study. Baseline steps were obtained by pedometer and categorized by tertiles (lower, middle, upper), while BMI was categorized into normal weight, overweight, and obesity. To explore joint associations, daily steps were dichotomized into “high steps” (middle/upper tertiles) and “low steps” (lower tertile), while BMI was dichotomized into normal weight and overweight/obesity. The joint exposure categories included “low steps and overweight/obesity,” “low steps and normal weight,” “high steps and overweight/obesity,” and “high steps and normal weight.”

**Results:**

We identified 35 (7.9%) cases of GERD over a mean follow-up of 2.5 years. Compared to the lower tertile of steps, the hazard ratios (HRs) [95% confidence intervals (95% CIs)] of GERD were 0.44 (0.20–0.96) and 0.17 (0.05–0.54) for the middle and upper tertiles, respectively, after adjusting for confounders (including BMI). Compared to normal weight, the HRs (95% CIs) of GERD were 1.35 (0.54–3.37) and 3.00 (1.19–7.55) for overweight and obesity, respectively, after adjusting for confounders (including steps). In a joint analysis, compared to “low steps and overweight/obesity,” the HRs (95% CIs) of GERD were 0.32 (0.10–1.00), 0.23 (0.10–0.54), and 0.20 (0.07–0.58) for “low steps and normal weight,” “high steps and overweight/obesity,” and “high steps and normal weight,” respectively.

**Conclusion:**

Higher daily steps were associated with a lower risk of GERD in older adults, independent of BMI. Since accumulating steps through walking is an achievable and acceptable modality of physical activity in older adulthood, future lifestyle interventions designed to achieve high daily steps counts may have favorable implications for the development of GERD in older adults of any BMI status.

## Introduction

Gastroesophageal reflux disease (GERD) is a multifaceted disorder of the upper gastrointestinal (GI) tract with a global prevalence of ∼13% (1). The long-term presence of GERD heightens the risk of adverse health outcomes such as difficulty swallowing (2), destruction of the esophageal lining (3), and esophageal cancer (4). While the interaction between multiple exposures (e.g., tobacco use, alcohol intake, genetic susceptibility, diet) likely underpins the development of GERD, high body mass index (BMI) and older age represent two of the most potent risk factors (1, 5). At present, approximately 40% of adults aged 60 and above are obese in the United States (6). By 2030, it is estimated that nearly 1 in 2 Americans will be obese (7) at which point the population of older adults will rise to 73 million (roughly 20% of the entire US population) (8), suggesting the prevalence and consequences of GERD will likely worsen with time.

Reducing excessive body mass, a modifiable risk factor of GERD, represents the cornerstone of GERD prevention (9). However, long-term intentional weight loss requires significant and often unsustainable changes to lifestyle behaviors (10), and weight reduction in older adulthood is further complicated by increased susceptibility to sarcopenia resulting from concomitant losses in lean body mass (11). Thus, elucidating strategies for mitigating the occurrence of GERD, independent of weight status, could have favorable implications for GERD development in older adulthood. Increased physical activity represents one such strategy (12), potentially working independently of weight change by improving the strength qualities of the diaphragm, or by promoting faster gastric emptying, both of which are implicated in the etiology of GERD (13). While a previous meta-analysis concluded that greater physical activity is likely associated with a lower prevalence of GERD in adulthood (13), all studies featuring in this analysis were limited by their cross-sectional/case-control design, self-reported indices of physical activity, and absence of joint exposure analyses. The present study provides a prospective exploration of the independent and joint associations of objectively-measured physical activity with incident GERD, thereby addressing the three primary limitations of previous epidemiologic studies: (1) susceptibility to reverse causation; (2) information bias caused by misclassification of physical activity; and (3) the lack of combined exposure phenotypes to provide a novel public health message about the joint associations of physical activity and BMI with GERD risk.

The assessment of daily steps has become an increasingly popular approach to capturing and promoting physical activity in older adulthood (14). Steps can be reliably measured using inexpensive pedometers or by harnessing smart phone applications (15), and step counts are an easily interpreted metric that older adults may favor over traditional time and intensity-based prescriptions of physical activity (16). Furthermore, a growing body of epidemiologic evidence suggests that greater daily steps are favorably associated with a wide range of outcomes in older adulthood, including activities of daily living (17), cardiovascular disease (18), and premature mortality (19). Step-based measures of physical activity therefore represent a cost-effective and acceptable alternative to other measurement indices of physical activity (e.g., accelerometry), and walking for the promotion of health is a major component of current physical activity guidelines for older adults (20), meaning step-based measures directly address a current public health need. However, the extent to which daily steps influence the risk of GERD in older adults is still poorly understood, and elucidating this relationship could positively impact public health guidance for the prevention of GERD by leveraging a metric of physical activity that is easily adoptable by older adults (16). Thus, the purpose of this study was to prospectively evaluate the independent and combined associations of pedometer-assessed daily steps and BMI with incident GERD in a cohort of older adults. We hypothesized that greater daily steps would be associated with a lower risk of GERD, independent of BMI. We anticipate that such novel findings will contribute to future public health strategies for mitigating the development of GERD in older adult population of all BMI statuses.

## Materials and methods

### Study population

The analytic sample was drawn from the “Physical Activity and Aging Study” (PAAS): a continuous enrollment, prospective cohort consisting exclusively of adults aged 65 years and above. Participants undergo a series of health and physical function assessments conducted over two visits, 1 week apart. Participants are then invited for follow-up visits on a yearly (or longer) basis, repeating the process until they move away from the local area, decide to withdraw, or become deceased. Recruitment into the study is done by word of mouth; targeted e-mailing to university faculty and staff; mass mailing of marketing material within the local area; and face-to-face recruitment at local organizations where senior citizens congregate (e.g., churches, voluntary organizations, retirement communities, etc.). At the time of analysis (April 2022), 584 individuals had at least one baseline and one follow-up visit with PAAS. Of these, we excluded 30 individuals because they had GERD at baseline, while a further 112 were excluded due to missing/invalid step count and/or covariate data, resulting in a final analytical sample of 442 individuals aged 65–91 years. The study was conducted in accordance with the Declaration of Helsinki, with participants providing written informed consent at each assessment visit. The PAAS protocol was initially approved by the ISU Institutional Review Board on August 25, 2015 (IRB ID: 15–430), renewal of which occurs on an annual basis.

### Assessment of daily steps and BMI

Participants were provided a tri-axial accelerometer-based pedometer (Omron HJ-321, Kyoto, Japan) and were instructed to wear the device on their hip or in their pocket during all waking hours for 7 continuous days (removing it only for water-based activities, bathing, and sleeping). Participants were also given a paper-based log to record their pedometer wear times during this period. We considered pedometer data to be valid if participants wore the device for at least 10 cumulative hours on at least 4 or more days a week, following prior studies (21, 22). Daily steps were calculated by dividing the sum of steps accumulated on valid days by the total number of valid days.

We grouped participants into the following tertiles (thirds) of daily steps: lower, middle, and upper, corresponding to <4,022, 4,022–6,662, >6,662 steps/day, respectively. By categorizing our primary exposure in such a manner, we are better positioned to convey an easily interpretable public health message about daily steps, BMI, and incident GERD. This is because risk estimates of GERD are made in relation to the “low step” reference group, illustrating how GERD risk changes when you move from one tertile to another tertile. We chose to use tertiles for this study to preserve statistical power by allowing adequate distribution of participants and cases of incident GERD across groups. The use of tertiles had two additional benefits for our study: (1) it catered for a more appropriate joint analyses with BMI, which was also an ordinal variable with three levels (normal weight, overweight, and obesity); and (2) it matched the three levels used by the alternative cut points of daily steps (another ordinal variable) derived from the step-based literature: <5,000, 5,000–7,499, and >7,499 steps/day (23). These alternative cut points are particularly relevant to public health because previous research has shown that mortality risk in older adults tapers off at ∼7,500 steps/day (21), meaning these thresholds are valid for our sample demographic.

Body mass and stature were measured using a digital scale (SECA, Model 769, Chino, CA, USA) and portable stadiometer, respectively. BMI was defined as body mass (kg) divided by height (meters squared). Participants were categorized into normal weight (<25 kg/m^2^), overweight (25 to <30 kg/m^2^), or obesity (≥30 kg/m^2^) using World Health Organization (WHO) cut points (24). To explore the joint associations of daily steps and BMI with incident GERD, we dichotomized daily steps into “low steps” (lower steps tertile) and “high steps” (middle and upper steps tertiles), and we dichotomized BMI into “normal weight” (<25 kg/m^2^) and “overweight/obesity” (≥25 kg/m^2^). We then created four combined steps/BMI categories: (1) “low steps and overweight/obesity” (reference), (2) “low steps and normal weight,” (3) “high steps and overweight/obesity,” and (4) “high steps and normal weight.” We also created four combined steps/BMI categories using the alternative cut-points of daily steps to provide additional evidence of the joint associations of steps and BMI with incident GERD. These categories included: (1) “<5,000 steps/day and overweight/obesity” (reference), (2) “<5,000 steps/day and normal weight,” (3) “≥5,000 steps/day and overweight/obesity,” and (4) “≥5,000 steps/day and normal weight”.

### Incident GERD

A physician diagnosis of GERD was captured by self-report using a standardized medical history questionnaire, a common approach to GERD identification in epidemiologic research (13). Participants were specifically asked: “*Have you ever been diagnosed with gastroesophageal reflux disease (GERD) by a physician?”* If the participant answered “*yes,”* then they additionally provided the year of first diagnosis, allowing for an accurate determination of the time course of our primary outcome. Participants with GERD at baseline were excluded from the analytic sample, therefore incident GERD was considered the first occurrence of the outcome after the baseline visit.

### Covariates

Information about covariates were extracted directly from responses to the medical history questionnaire or by using objectively assessed indices from the health assessments at baseline. The covariates chosen for this analysis were based on prior knowledge of the scientific literature, with their selection seeking to minimize biased estimates of the association between the exposures of interest (i.e., daily steps and BMI) and the primary outcome (incident GERD), of which the following were included in regression models: age (years); sex (male or female); hypertension (yes or no); history of cardiovascular disease [CVD], i.e., myocardial infarction, congestive heart failure, or stroke (yes or no); history of cancer [excluding skin cancer] (yes or no); diabetes [type 1 or type 2] (yes or no); smoking status (never, former, or current), asthma (yes or no); heavy drinking (yes or no), hypercholesterolemia (yes or no); fruit intake (cups/day), and vegetable intake (cups/day). Hypertension was captured by the self-reported physician diagnosis, self-reported use of hypertension medication, or a resting systolic and/or diastolic blood pressure of ≥130/80 mmHg. Diabetes was captured by self-reported physician diagnosis, self-reported use of diabetes medication, or a fasting blood glucose concentration ≥126 mg/dl. Asthma was captured by self-reported physician diagnosis or by self-reported use of asthma medication. Hypercholesterolemia was captured by self-reported physician diagnosis, self-reported use of cholesterol medication (including statins), or a low-density lipoprotein cholesterol concentration ≥160 mg/dl. Heavy drinking was defined as an average of >14 drinks/week and >7 drinks/week for males and females, respectively (25).

### Statistical analysis

Baseline characteristics are presented by categories of daily steps and BMI. Groups were compared using general linear models for continuous variables or chi squared (*χ*^2^) tests for categorical variables. We used Cox proportional hazard models to estimate the hazard ratios (HRs) and 95% confidence intervals (95% CIs) of incident GERD across categories of daily steps (tertiles and alternative cut-points) and categories of BMI, while adjusting for potential confounders. Cumulative hazard plots grouped by the main exposures (i.e., tertiles of daily steps, categories of steps using the alternative cut points, and categories of BMI) showed no significant violations of the proportional hazard assumption.

Our basic Cox regression model (Model 1) simply adjusted for age and sex (demographic variables), while Model 2 additionally adjusted for covariates of health behavior/health status known to influence the exposures and/or outcome (hypertension, history of CVD, history of cancer, diabetes, smoking status, asthma, heavy alcohol drinking, hypercholesterolemia, cups/day of fruit [quintiles], and cups/day of vegetables [quintiles]) (26). Model 3 catered for the evaluation of the independent association of daily steps or BMI with incident GERD by mutually adjusting for each in separate models [i.e., BMI (kg/m^2^) in the daily steps analysis, or daily steps in the BMI analysis]. We also estimated the HRs (95% CIs) of incident GERD per 1,000 step increase in daily steps, and per one unit increase in BMI using the continuous measure of each variable, catering for an easily translatable public health message about the change in GERD risk for each realistic increase in step counts or BMI. In the joint analyses, the “low steps and overweight/obesity” group served as the referent, and we adjusted for the same covariates listed in Model 2 above.

We also performed a stratified analysis to evaluate the risk of GERD across various subgroups of the sample population. Here, the HRs (95% CIs) of incident GERD were calculated as a function of high daily steps (middle and upper tertile of daily steps) vs. the referent of low daily steps (lower tertile of daily steps). The sub groups evaluated included sex, age (<75 or ≥75 years), BMI (normal weight, overweight, obesity), hypertension (yes or no), history of cancer (yes or no), hypercholesterolemia (yes or no), smoking status (former/current or never), and meeting the national intake guidelines of fruit (≥1.5 cups/day) and vegetables (≥2 cups/day) as per the recommendations of the United States Department of Agriculture (27). Stratification based on the other covariates listed above (i.e., history of CVD, diabetes, asthma, and heavy drinking) was not undertaken due to low case numbers of GERD in these strata.

All analyses were conducted using SAS version 9.4 (SAS Institute, Inc., Cary, NC), and we considered a two-sided *p*-value <0.05 to be significant.

## Results

There were 35 (7.9%) incident cases of GERD reported over a mean (SD) follow-up time of 2.5 (1.9) years. Table 1 shows the participant characteristics at baseline by tertiles of daily steps and categories of BMI. Significant differences in baseline characteristics between the lower, middle, and upper tertiles of daily steps were found for the following: age (mean [SD] years: 74.4 [6.5], 71.8 [5.5], and 70.1 [4.5], respectively); heavy drinking status (frequency [SD]: 4 [2.7], 15 [10.1], and 8 [5.4], respectively); prevalent diabetes (frequency [%]: 20 [13.6], 6 [4.1], and 9 [6.1] respectively); and vegetable intake (mean [SD] cups/day: 1.9 [2.9], 1.8 [1.1], and 3.1 [7.1], respectively). Significant differences in baseline characteristics between normal weight, overweight, and obesity categories were found for the following: age (mean [SD] years: 72.9 [6.1], 72.1 [5.9], and 70.8 [5.0], respectively); female sex (frequency [%]: 128 [72.3], 87 [52.4], and 43 [43.3], respectively); prevalent hypertension (frequency [%]: 89 [50.3], 102 [61.5], and 69 [69.7], respectively); and prevalent diabetes (frequency [%]: 6 [3.4], 13 [7.8], and 16 [16.2], respectively). Baseline characteristics by cases and non-cases of GERD can be found in Supplementary Table S1.

**Table 1:** Baseline characteristics of participants by categories of daily steps and body mass index.

Characteristic		Tertile of daily steps				Body mass index (BMI)			
	All	Lower	Middle	Upper	*P-*value^a^	Normal weight	Overweight	Obesity	*P*-value^a^
		(<4,022 steps/day)	(4,022–6,662 steps/day)	(>6,662 steps/day)		(<25.0 kg/m^2^)	(25.0 to <30 kg/m^2^)	(≥30 kg/m^2^)	
Number	442	147	148	147	—	177	166	99	—
Age (years)	72.1 (5.8)	74.4 (6.5)	71.8 (5.5)	70.1 (4.5)	**<0** **.** **001**	72.9 (6.1)	72.1 (5.9)	70.8 (5.0)	**0** **.** **015**
Female, *n* (%)	258 (58.4)	93 (63.3)	84 (56.8)	81 (55.1)	0.324	128 (72.3)	87 (52.4)	43 (43.3)	**<0** **.** **001**
Daily steps (steps/day)	5,866 (3,278)	2,849 (836)	5,159 (777)	9,595 (2,733)	**<0** **.** **001**	6,056 (3,187)	5,882 (3,357)	5,500 (3,306)	0.401
BMI (kg/m^2^)	26.8 (4.6)	27.4 (5.1)	26.9 (4.2)	26.3 (4.4)	0.111	22.7 (1.7)	27.3 (1.4)	33.5 (3.3)	**<0** **.** **001**
Never smoker, *n* (%)	310 (70.1)	105 (71.4)	104 (70.3)	101 (68.7)	0.644	122 (68.9)	120 (72.3)	68 (68.7)	0.700
Former smoker, *n* (%)	129 (29.2)	40 (27.2)	43 (29.1)	46 (31.3)		53 (29.9)	46 (27.7)	30 (30.3)	
Current smoker, *n* (%)	3 (0.7)	2 (1.4)	1 (0.7)	0		2 (1.1)	0	1 (1.01)	
Heavy alcohol drinking^b^, *n* (%)	27 (6.1)	4 (2.7)	15 (10.1)	8 (5.4)	**0** **.** **027**	12 (6.8)	11 (6.6)	4 (4.0)	0.620
Hypertension^c^, *n* (%)	260 (58.8)	89 (60.5)	90 (60.8)	81 (55.1)	0.532	89 (50.3)	102 (61.5)	69 (69.7)	**0** **.** **005**
History of CVD^d^, *n* (%)	21 (4.8)	8 (5.4)	7 (4.7)	6 (4.1)	0.860	10 (5.7)	8 (4.8)	3 (3.0)	0.617
History of cancer^e^, *n* (%)	110 (24.9)	45 (30.6)	36 (24.3)	29 (19.7)	0.096	36 (20.3)	41 (24.7)	33 (33.3)	0.057
Diabetes^f^, *n* (%)	35 (7.9)	20 (13.6)	6 (4.1)	9 (6.1)	**0** **.** **006**	6 (3.4)	13 (7.8)	16 (16.2)	**0** **.** **007**
Hypercholesterolemia^g^, *n* (%)	228 (51.6)	72 (49.0)	73 (49.3)	83 (56.5)	0.350	86 (48.6)	87 (52.4)	55 (55.6)	0.520
Asthma^h^, *n* (%)	26 (5.9)	8 (5.4)	10 (6.8)	8 (5.4)	0.858	6 (3.4)	15 (9.0)	5 (5.1)	0.078
Fruit intake (cups/day)	2.2 (5.1)	2.0 (3.3)	1.8 (1.6)	2.8 (8.0)	0.180	2.2 (3.2)	2.5 (7.6)	1.7 (1.2)	0.480
Vegetable intake (cups/day)	2.2 (4.5)	1.9 (2.9)	1.8 (1.1)	3.1 (7.1)	**0** **.** **028**	2.5 (4.8)	2.3 (5.3)	1.8 (1.1)	0.544

Data are presented as mean (SD) or *n* (%). CVD, cardiovascular disease.

^a^ *P*-value for the comparison between categories of daily steps or categories of BMI: *χ*^2^ (categorical) or general linear model *F*-tests (continuous).

^b^ Heavy alcohol drinking defined as >7 alcoholic drinks/week for females or >14 alcoholic drinks/week for males.

^c^ Defined as systolic/diastolic blood pressure ≥130/80 mmHg, self-reported physician diagnosis of hypertension, and/or taking blood pressure medication.

^d^ Defined as self-reported physician diagnosis myocardial infarction, stroke, and/or congestive heart failure.

^e^ Defined as self-reported physician diagnosis of cancer (except skin cancer).

^f^ Defined as blood glucose concentration >126 mg/dl, self-reported physician diagnosis of diabetes (type 1 or 2), and/or taking diabetes medication.

^g^ Defined self-reported physician diagnosis, self-reported use of cholesterol medication (including statins), or a low-density lipoprotein cholesterol concentration ≥160 mg/dl.

^h^ Defined as self-reported physician diagnosis of asthma and/or taking asthma medication.

Bolded values indicate *P *< 0.05.

Cases (%) of incident GERD for the lower, middle, and upper tertiles of daily steps were 20 (13.6), 11 (7.4), and 4 (1.7) (*p-*value <0.001). Compared to those in the lower tertile of daily steps, the HRs (95% CIs) of incident GERD for the middle and upper tertiles of daily steps were 0.44 (0.20–0.96) and 0.17 (0.05–0.54), respectively (*P* for linear trend = 0.001), after adjusting for potential confounders including BMI (Table 2). Cases (%) of incident GERD when using the alternative cut points of daily steps were 28 (12.8), 3 (2.7), and 4 (3.6) for <5,000 steps/day, 5,000–7,499 steps/day, and ≥7,500 steps/day, respectively. Compared to those who amassed <5,000 steps/day, the HRs (95% CIs) of incident GERD 0.18 (0.05–0.61) and 0.24 (0.07–0.73) for those who amassed 5,000–7,499 steps/day and ≥7,500 steps/day, respectively (*P* for linear trend = 0.002), after adjusting for potential confounders including BMI (Table 2). The HR (95% CI) of incident GERD for each additional 1,000 steps was 0.78 (0.66–0.92) in the fully adjusted model, suggesting a 22% lower risk of GERD (Table 2). Cases (%) of incident GERD for the normal weight, overweight, and obesity categories were 9 (5.1), 11 (6.6), and 15 (15.2), respectively. Compared to normal weight, the HRs (95% CIs) of incident GERD were 1.35 (0.54–3.37) and 3.00 (1.19–7.55) for overweight and obesity, respectively (*P* for linear trend = 0.019), after adjusting for potential confounders including daily steps (Table 2). The HR (95% CI) of incident GERD per unit BMI was 1.08 (0.99–1.17) in the fully adjusted model, suggesting an 8% greater risk of GERD, though this association was not significant (Table 2).

**Table 2:** Hazard ratios (HRs) and 95% confidence intervals (CIs) of incident gastroesophageal reflux disease (GERD) by categories of daily steps and body mass index (BMI).

Tertiles	*n*	Cases (%) of GERD	HR (95% CI)		
			Model 1^a^	Model 2^b^	Model 3^c^
Lower: <4,022 steps/day	147	20 (13.6)	1.00 (reference)	1.00 (reference)	1.00 (reference)
Middle: 4,022–6,662 steps/day	148	11 (7.4)	**0.47** **(0.22–0.99)**	**0.43** **(0.20–0.93)**	**0.44** **(0.20–0.96)**
Upper: >6,662 steps/day	147	4 (2.7)	**0.16** **(0.05–0.49)**	**0.16** **(0.05–0.50)**	**0.17** **(0.05–0.54)**
*P* for linear trend			**<0.001**	**<0.001**	**0.001**
Alternative cut points	*n*	Cases (%) of GERD	Model 1^a^	Model 2^b^	Model 3^c^
<5,000 steps/day	219	28 (12.8)	1.00 (reference)	1.00 (reference)	1.00 (reference)
5,000–7,499 steps/day	111	3 (2.7)	**0.20** **(0.06–0.68)**	**0.17** **(0.05–0.60)**	**0.18** **(0.05–0.61)**
≥7,500 steps/day	112	4 (3.6)	**0.23** **(0.08–0.68)**	**0.22** **(0.07–0.65)**	**0.24** **(0.07–0.73)**
*P* for linear trend			**0.002**	**0.001**	**0.002**
Per 1,000 increase in steps/day			**0.78** **(0.67–0.91)**	**0.77** **(0.65–0.90)**	**0.78** **(0.66–0.92)**
BMI	*n*	Cases (%) of GERD	Model 1^a^	Model 2^b^	Model 3^c^
Normal weight: <25 kg/m^2^	177	9 (5.1)	1.00 (reference)	1.00 (reference)	1.00 (reference)
Overweight: 25 to <30 kg/m^2^	166	11 (6.6)	1.43 (0.59–3.49)	1.37 (0.55–3.41)	1.35 (0.54–3.37)
Obesity: ≥30 kg/m^2^	99	15 (15.2)	**3.51** **(1.47–8.40)**	**3.57** **(1.43–8.91)**	**3.00** **(1.19–7.55)**
*P* for linear trend			**0.005**	**0.006**	**0.019**
Per 1 unit increase in BMI			**1.09** **(1.02–1.17)**	**1.10** **(1.02–1.19)**	**1.08** **(0.99–1.17)**

^a^ Model 1 adjusted for age (years), sex (male or female).

^b^ Model 2 adjusted for Model 1 plus hypertension (yes or no), history of CVD (yes or no), diabetes (yes or no), hypercholesterolemia (yes or no), asthma (yes or no), history of cancer (yes or no), smoking status (never, former, current), heavy alcohol drinking (yes or no), cups per day of fruit (quintiles), and cups per day of vegetables (quintiles).

^c^ Model 3 adjusted for Model 2 plus body mass index [BMI] (kg/m^2^) for the steps analysis or steps/day for the BMI analysis.

Bolded values indicate *P *< 0.05.

**Figure 1:**
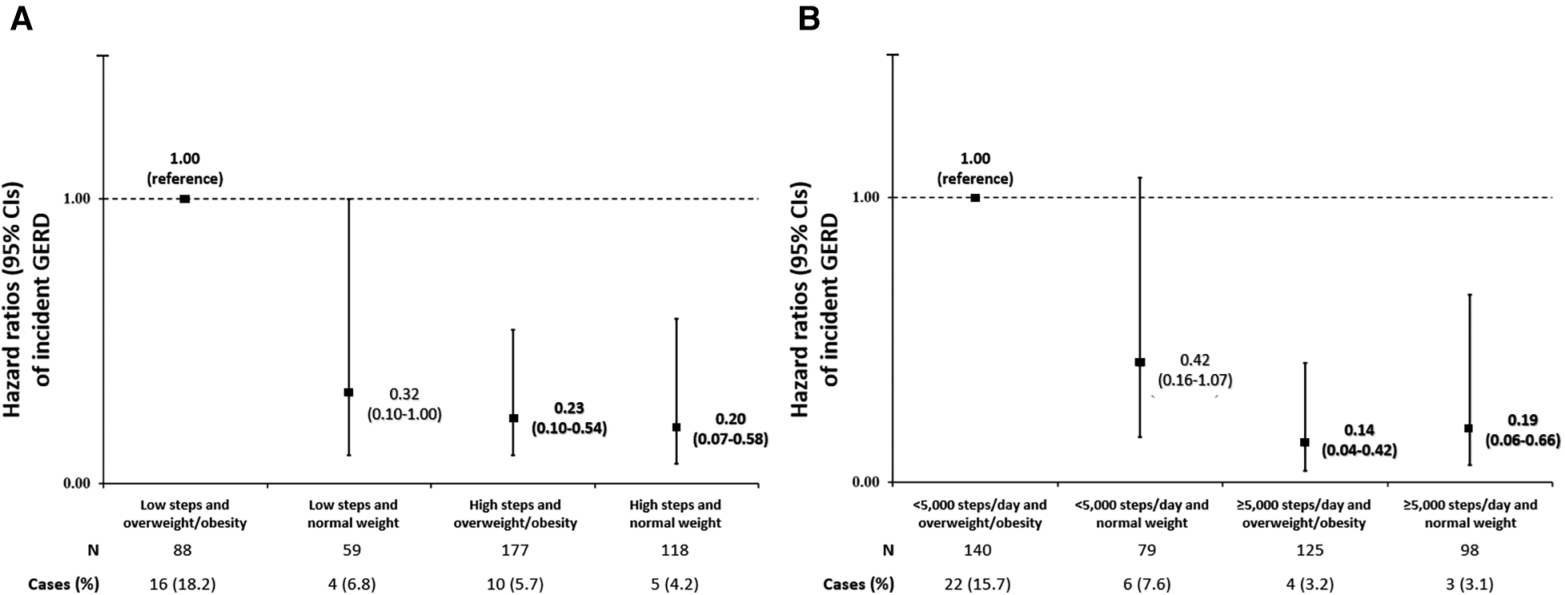
(**A,B**) Joint associations of daily steps and body mass index (BMI) with incident gastroesophageal reflux disease (GERD) using either the tertile distribution of daily steps (**A**) or the alternative cut points of steps (**B**) participants were divided into four groups based on combined categories of daily steps (“high steps” or “low steps”) and body mass index (normal weight or overweight/obesity). In (**A**), “high steps” were defined as the middle and upper tertiles of daily steps, while “low steps” were defined as the lower tertile of daily steps. In (**B**), participants were divided into four groups based on the alternative cut points of daily steps (“high steps”: ≥5,000 steps/day, “low steps”: <5,000 steps/day). In both (**A,B**), normal weight was defined as BMI <25.0 kg/m^2^, while overweight/obesity was defined as ≥25 kg/m^2^. We used Cox proportional hazards regression to estimate the association of these joint exposures with incident GERD, adjusting for sex (male of female), age (years), hypertension (yes or no), history of CVD (yes or no), diabetes (yes or no), hypercholesterolemia (yes or no), asthma (yes or no), history of cancer (yes or no), current smoking status (never, former, or current), heavy alcohol drinking (yes or no), cups/day of fruit (quintiles), and cups/day of vegetables (quintiles). Bolded values indicate *p *< 0.05.

**Figure 2:**
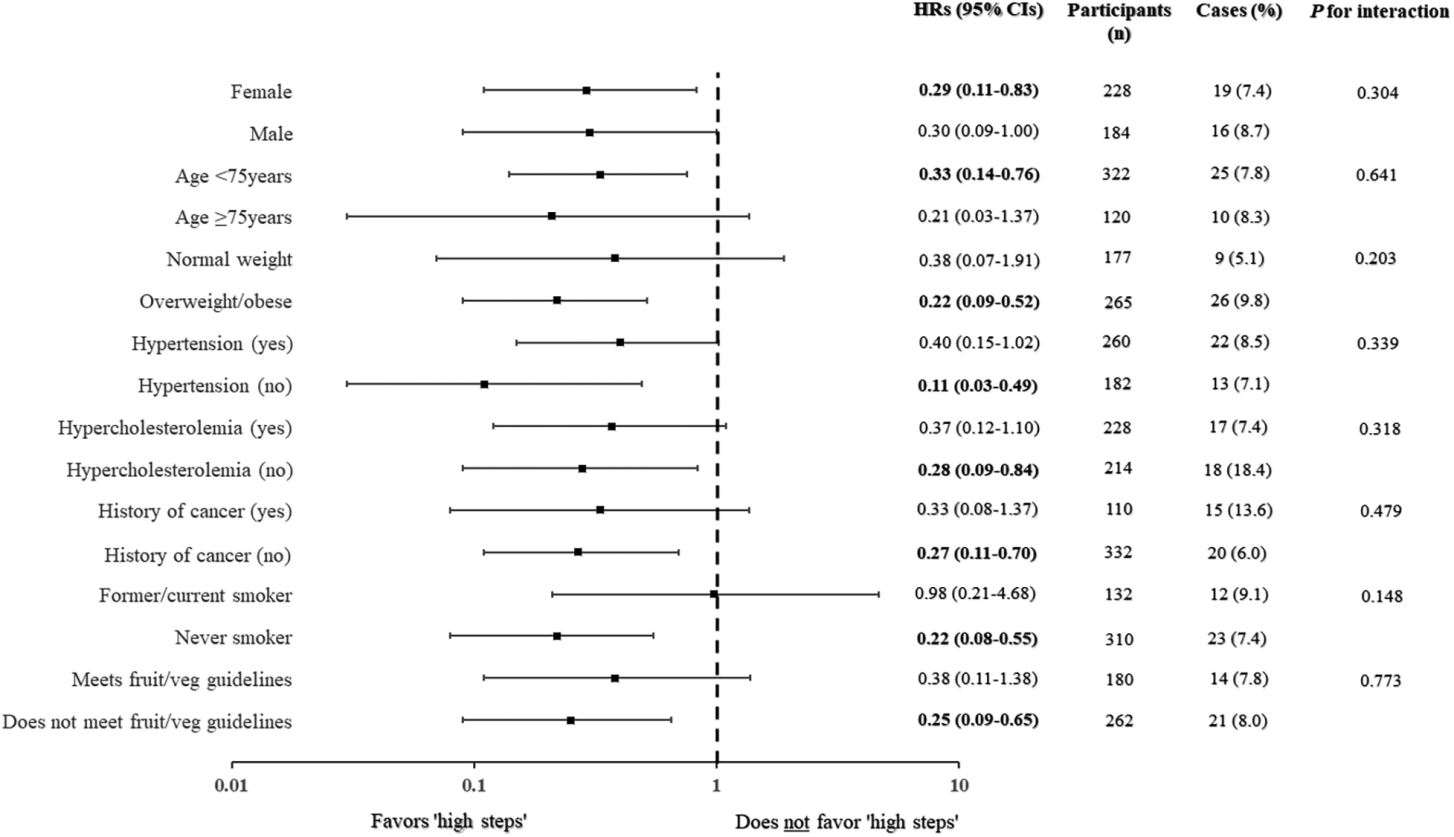
Hazard ratios (HRs) and 95% confidence intervals (95% CI) of incident gastroesophageal reflux disease (GERD) based on daily steps among various subgroups. HRs are depicted by the black squares and 95% CIs by the whiskers. The reference group for all analyses was the “low steps” group (lower tertile of daily steps). The Cox proportional hazards regression models were adjusted for sex (not in sex-stratified analysis), age (not in age-stratified analysis), BMI (not in BMI-stratified analysis), hypertension (not in hypertension-stratified analysis), history of CVD, diabetes, hypercholesterolemia (not in hypercholesterolemia-stratified analysis), asthma, history of cancer (not in cancer-stratified analysis), current smoking status (not in smoking-stratified analysis), heavy alcohol drinking, cups/day of fruit (quintiles) (not in fruit/veg-stratified analysis), and cups/day of vegetables (quintiles) (not in fruit/veg-stratified analysis). Bolded values indicate *p *< 0.05.

In our first joint analysis, where tertiles of daily steps were combined with categories of BMI, the cases (%) of incident GERD were as follows: 16 (18.2), 4 (6.8), 10 (5.7), and 5 (4.2) for those categorized into the “low steps and overweight/obesity,” “low steps and normal weight,” “high steps and overweight/obesity,” and “high steps and normal weight groups,” respectively. Compared to the referent group of “low steps and overweight/obesity,” the adjusted HRs (95% CIs) of incident GERD were 0.32 (0.10–1.00), 0.23 (0.10–0.54), and 0.20 (0.07–0.58) for the “low steps and normal weight,” “high steps and overweight/obesity,” and “high steps and normal weight” groups, respectively (Figure 1A). In the second joint analysis, where the alternative cut points for daily steps were combined with categories of BMI, the cases (%) of incident GERD were as follows: 22 (15.7), 6 (7.6), 4 (3.2), and 3 (3.1) for those categorized into the “<5,000 steps/day and overweight/obesity,” “5,000 steps/day and normal weight,” “≥5,000 steps/day and overweight/obesity,” and “≥5,000 steps/day and normal weight groups,” respectively. Compared to the referent group of “<5,000 steps/day and overweight/obesity,” the adjusted HRs (95% CIs) of incident GERD were 0.42 (0.16–1.07), 0.14 (0.04–0.42), and 0.19 (0.06–0.66) for the “<5,000 steps/day and normal weight,” “≥5,000 steps/day and overweight/obesity,” and “≥5,000 steps/day and normal weight” groups, respectively (Figure 1B). Finally, in our stratified analyses (Figure 2), being categorized into the higher daily steps phenotype (i.e., middle and upper tertiles of daily steps) was associated with a lower risk of GERD across all subgroups, with significant associations found for female sex (HR [95% CI]: 0.29 [0.11–0.83]); those aged <75 years (HR [95% CI]: 0.33 [0.14–0.76]); overweight/obesity (HR [95% CI]: 0.22 [0.09–0.52]); those without hypertension (HR [95% CI]: 0.11 [0.03–0.49]); those without hypercholesterolemia (HR [95% CI]: 0.28 [0.09–0.84]); those without a history of cancer (HR [95% CI]: 0.27 [0.11–0.70]); never smokers (HR [95% CI]: 0.22 [0.08–0.55]); and those who did not meet the US guidelines for weekly fruit and vegetable and intake (HR [95% CI]: 0.25 [0.09–0.65]).

## Discussion

This study found that greater daily steps were significantly associated with a lower risk of incident GERD, independent of BMI. We additionally found that obesity, but not overweight, was significantly associated with an increased risk of incident GERD after adjusting for potential confounders, including daily steps. Our two joint analyses further suggest that a “high steps” phenotype (categorized as either middle/upper tertile of daily steps or ≥5,000 steps/day using the alternative cut points) combined with any category of BMI (normal weight or overweight/obesity) was associated with significantly lower risks of incident GERD when compared to those with a “low steps and overweight/obesity” phenotype. Lastly, our stratified analyses demonstrated consistency in the association between high daily steps and incident GERD across many subgroups, including the high-risk phenotype of overweight/obesity. Our data provides a unique contribution to the scientific literature as the first prospective study to evaluate the independent and combined associations of objectively assessed daily steps and BMI with incident GERD in older adults, a subsection of the broader adult population that are at greatest risk of developing GERD. Together, these findings infer that amassing high daily steps (i.e., >4,000 steps/day) may mitigate GERD risk independent of person's BMI status. These are novel findings that have real-world implications for public health policy makers seeking to develop cost-effective strategies for minimizing GERD development in older adults of any BMI status.

Several prospective studies have explored the associations between daily steps and a range of health outcomes in older adults, including depressive symptoms (28), dementia (29), diabetes (30), CVD (18), cancer (31), and mortality (19). These studies demonstrate that, after accounting for potential confounders, the accumulation of greater daily steps is associated with lower risks of common age-related morbidities and premature death, and the present study is broadly consistent with this paradigm. Our findings also align with the only other study to have explored objectively assessed daily steps and incident GERD in an aging population. In their analysis of 6,042 adults (median age 56.7 years) followed over median period of 4 years, Master and colleagues found that compared to the 25th percentile (≤6,140 steps/day) of daily steps, those in the 75th percentile (≥10,760 steps/day) had a 29% lower risk of GERD after adjusting for potential confounders, including BMI (32). However, unlike the present study, Master and colleagues did not evaluate the joint associations of daily steps and BMI with incident GERD. Our findings therefore have novel implications for public health beyond the initial findings of Master and colleagues because the present study suggests that higher steps are associated with a lower risk of GERD even when combined with an overweight/obesity phenotype.

High BMI is a potent and well-established risk factor of GERD, and global estimates indicate those with obesity are 1.73 times more likely to have GERD than individuals with normal weight (1). In the present study, the incidence of GERD was 5.1%, 6.6%, and 15.2% among those with normal weight, overweight, and obesity phenotypes, respectively. When compared to normal weight, the risk of GERD was 1.35 and 3 times greater for those with the overweight and obesity phenotypes, respectively, after adjusting for potential confounders (including daily steps). These data are consistent with our current understanding of the dose response relationship between BMI and GERD risk (33). Significant linear trends (*P *< 0.05) in the risk of this morbidity were observed across all three categories of BMI in the present study, and the obesity category remained significantly associated with GERD even after adjustment for daily steps. While the observation that obesity is strongly associated with GERD in older adults is well-known, the fact that the association was not fully attenuated when adjusting for daily steps might otherwise suggest that obesity and incident GERD are independent of stepping behavior. However, we argue that this finding should not deter public health policy makers for promoting walking in older adults with obesity since any increase in physical activity behavior is likely to positively affect cardiometabolic risk factors of GERD (e.g., high blood pressure, high blood glucose, high total cholesterol) (34), and therefore result in wider health benefits. Nevertheless, further studies with larger sample sizes and longer follow-up times are needed to support the consistency of the association between obesity and incident GERD in older adults, independent of daily steps.

The mean (SD) daily steps for the normal weight and the obesity categories in the present study were 6,056 (3,187) and 5,500 (3,306), respectively. Furthermore, a similar proportion of participants in either BMI category had “high steps” (≥4,022 steps/day): 72% and 65% of participants in the normal weight and obesity categories, respectively. Given that both phenotypes had similar average daily steps with a similar proportion of “high steppers,” it may be that the modifying influence of daily steps on GERD differs between obesity and non-obesity phenotypes, perhaps due to unmeasured confounders that would be expected to differ between overweight and obesity phenotypes (e.g., total energy intake). However, larger prospective studies with higher case numbers of GERD and more diverse measures of dietary intake are needed to evaluate the robustness of this claim.

Our joint analyses (Figures 1A,B) suggest that “high steps” of any BMI status (normal weight and/or overweight/obesity) is significantly associated with lower risks of GERD compared to those categorized as “low steps and overweight/obesity”. The first of these analyses (Figure 1A) dichotomized “low steps” and “high steps” using the lower tertile of daily steps and middle/upper tertiles of daily steps, respectively, while the second analyses dichotomized daily steps using the alternative cut points of daily steps: <5,000 steps/day and ≥5,000 steps/day to designate “low steps” and “high steps,” respectively (Figure 1B). Across the two different approaches, individuals categorized as “high steps and overweight/obesity” had between 77% and 86% lower risks of GERD compared to individuals with “low steps and overweight/obesity”. These novel data support the notion that higher daily steps moderate the risk of GERD attributed to high BMI in older adults. From a public health perspective, the implication of this finding is that older adults with overweight/obesity seeking to ameliorate their risk of GERD should consider the merits of accumulating more daily steps (i.e., >4,000 steps/day). Future interventions may additionally seek to target walking as a therapeutic strategy for combating GERD risk in older adults with overweight or obesity, though the feasibility of such an approach would need careful consideration given the latency of GERD development (35). It must also be noted that since we combined individuals with overweight or obesity into a single grouping to maintain statistical power, we were subsequently unable to evaluate the specific joint association of “high steps” and the standalone category of obesity with incident GERD. It is therefore possible that prospective studies with larger sample sizes and higher cases of GERD may find differential estimates of risk between overweight “high steppers” and “high steppers” with obesity.

The potential mechanisms by which higher daily steps attenuate the risk of GERD in older adults are not fully understood. It is widely asserted that greater levels of physical activity (e.g., walking) assist with achieving or maintaining a healthy body weight (36), which in turn could shield against the large intragastric pressures that drive the development of GERD in obesity (37). The maintenance of a healthy body weight through higher daily steps may partly explain the variability in the incidence of GERD between the obesity and normal weight phenotypes in the present study (15.2% vs. 5.1%, respectively), where those with normal weight averaged >500 steps/day more than their counterparts with obesity. Nevertheless, the overall findings of the present study are somewhat contradictory to this assertion, namely that higher daily steps were associated with lower risks of GERD independent of BMI. Higher daily steps may influence the functional qualities of the diaphragm (13), a structure of the lower esophageal region that functions as an anti-reflux barrier by influencing the tone of the lower esophageal sphincter (38). Older adults may be particularly susceptible to reflux due to age-associated weakening of the diaphragm (39), and engagement in regular physical activity could feasibly promote the strength qualities of this structure. However, randomized controlled trials exploring the causal link between physical activity and improved functioning of the anti-reflux barrier are lacking. Aging is also associated with slower rates of gastric emptying (40), and delays in the movement of stomach contents to the small intestine promote reflux (41). Limited evidence suggests that low-to-moderate intensity physical activity such as walking are associated with faster gastric emptying in adults with obesity (42), which may partly explain the independent association between daily steps and incident GERD in the present study.

Our findings must be interpreted in the context of several limitations. First, misclassification of our primary outcome may have biased our results toward the null given that GERD was obtained via self-report and not by objective measures (e.g., endoscopy). However, GERD is often confirmed via self-report in the primary care setting prior to any confirmatory testing (43), and our approach to capturing cases of GERD using a medical history questionnaire aligns with the approach taken by other large prospective studies (44, 45). Second, dietary habits such as total fat consumption and total energy intake are established risk factors of GERD (46). However, our analyses could not account for dietary risk factors other than self-reported fruit and vegetable consumption, meaning the associations found herein are susceptible to residual confounding. Third, the majority of participants were relatively healthy, highly educated, and independently living, meaning our findings have limited generalizability to wider older adult populations that are less healthy, less educated, and/or residing in assisted-living communities. Fourth, causal inferences are hindered by the observational nature of the study, though intervention studies such as randomized controlled trials would be difficult to conduct in the context of GERD given the latency in the development of this morbidity (35), as well as the ethical implications of withholding a treatment (i.e., physical activity) that may potentially prevent disease occurrence (47). Finally, given the present study's limited sample size (*n* = 442) and relatively homogenous demographic composition, it is feasible that the association of daily steps and BMI with incident GERD was underestimated. Indeed, the incidence of GERD in the present study was only 7.9%, and the proportion of non-Hispanic whites was >95%. Current estimates suggest GERD prevalence ranges from 18%–28% in North America (48), and the condition extends beyond non-Hispanic whites (49). Thus, larger sample sizes composed of racially and ethnically diverse older adult participants could maximize the generalizability of future studies.

Despite these limitations, our study is one of only a few prospective analyses of daily steps with incident GERD, and it is currently the only study to evaluate both the independent and joint associations of daily steps and BMI (a well-established risk factor of GERD) with the incidence of this morbidity in older adults. Our data therefore fills a key knowledge gap in the academic literature, emphasizing how a greater accumulation of daily steps attenuates the risk of GERD, independent of BMI, in a population at greatest risk of developing GERD. This, in turn, provides evidence for public health policy makers seeking to develop cost-effective and easily adopted strategies for minimizing GERD risk in older adult populations. Another strength includes the assessment of free-living stepping behavior using a low-cost pedometer to objectively capture daily step counts, reducing the effects of information bias caused by inaccurate recalling of self-reported physical activity (a major limitation of previous studies). Finally, the categorization of daily steps using alternative (literature-derived) cut-points, alongside an extensive subgroup analysis to evaluate the risk of GERD among various strata of our sample, highlights the consistency of the observed associations between daily steps and incident GERD in our sample of older adults.

In conclusion, this study demonstrated significant, inverse associations between daily steps and incident GERD, independent of BMI, the results of which were consistent across various subgroups. Our data also indicated that obesity, but not overweight, was associated with a significantly greater risk of incident GERD, even after adjustment for daily steps. This suggests that the relationship between obesity and incident GERD is potentially independent of daily stepping behavior, though additional studies are needed to evaluate the robustness of this finding. Finally, our two separate joint analyses showed that “high steps” (defined as the middle and upper tertile of the daily step distribution, or simply as achieving ≥5,000 steps/day) combined with any BMI phenotype (either normal weight or overweight/obesity) was associated with significantly lower risks of incident GERD compared to the joint category of “low steps and overweight/obesity.” This novel finding places emphasis on the relative importance of daily stepping behavior in the development of GERD among the high-risk older adult population with overweight and/or obesity. Future prospective studies with larger sample sizes, longer follow-up times, and with more racially and socioeconomically diverse populations are needed to support the consistency of our findings, thereby confirming generalizability to the wider older adult population.

Future studies may additionally seek to explore the independent associations of stepping cadence with incident GERD, perhaps determining which stepping behavior is more influential on GERD development: step volume or step intensity. Step-prescribed intervention studies should be conducted to evaluate the effects of increasing daily stepping behavior on important outcomes and/or comorbidities of GERD such as heartburn frequency, arterial hypertension, hypercholesterolemia, depressive symptoms, and constipation (50). Since greater physical activity is reasonably expected to be inversely associated with these outcomes, and since step-prescribed interventions in older adults with GERD are lacking, such an undertaking would address a critical knowledge gap in the literature while utilizing a novel and feasible research design.

## Data Availability

The datasets presented in this article are not readily available because it is owned by Iowa State University. However, legitimate researchers can request access to this data by submitting a request to our ethics board. Requests to access the datasets should be directed to PAAS@iastate.edu.
