# Supplementary material for: The associations of daily steps and body mass index with incident gastroesophageal reflux disease in older adults
Source: Front Sports Act Living. 2024 Apr 5;6:1384845. doi: 10.3389/fspor.2024.1384845 (PMC11026570; doi:10.3389/fspor.2024.1384845)
Supplement: Supplementary file 1 [file Table1.docx]

Supplementary Material

**S1 Table. Baseline characteristics of participants by cases and non-cases of gastroesophageal reflux disease.**

| **Characteristic** | **All**  (n = 442) | **Cases**  (n = 35) | **Non-cases**  (n = 407) | ***P-value****^a^* |
| --- | --- | --- | --- | --- |
| **Age in years, mean (SD)** | 72.1 (5.8) | 72.3 (5.8) | 72.1 (5.8) | 0.876 |
| **Female, *N* (%)** | 258 (58.4) | 19 (54.3) | 239 (58.7) | 0.609 |
| **Daily step counts, mean (SD)** | 5866 (3278) | 4308 (2168) | 6000 (3324) | **0.003** |
| **BMI, mean (SD)** | 26.8 (4.6) | 28.3 (4.4) | 26.7 (4.6) | **0.049** |
| **Normal weight**^b^, ***N* (%)** | 177 (40.0) | 9 (25.7) | 168 (41.3) | **0.009** |
| **Overweight**^b^, ***N* (%)** | 166 (26.2) | 11 (31.4) | 155 (38.1) |  |
| **Obese**^b^, ***N* (%)** | 99 (22.4) | 15 (42.9) | 84 (20.6) |  |
| **Never smoker, *N* (%)** | 310 (70.1) | 23 (65.7) | 287 (70.5) | 0.704 |
| **Former smoker, *N* (%)** | 129 (29.2) | 12 (34.3) | 117 (28.8) |  |
| **Current smoker, *N* (%)** | 3 (0.7) | 0 | 3 (0.7) |  |
| **Heavy alcohol drinking^c^, *N* (%)** | 27 (6.1) | 1 (2.9) | 26 (6.4) | 0.403 |
| **Hypertension^d^, *N* (%)** | 260 (58.8) | 22 (62.9) | 238 (58.5) | 0.613 |
| **History of CVD^e^, *N* (%)** | 21 (4.8) | 2 (5.7) | 19 (4.7) | 0.780 |
| **History of cancer^f^, *N* (%)** | 110 (24.9) | 15 (42.9) | 95 (23.3) | **0.010** |
| **Diabetes^g^, *N* (%)** | 35 (7.9) | 2 (5.7) | 33 (8.1) | 0.615 |
| **Hypercholesterolemia^h^, *N* (%)** | 228 (51.6) | 17 (48.6) | 211 (51.8) | 0.710 |
| **Asthma^i^, *N* (%)** | 26 (5.9) | 3 (8.6) | 23 (5.7) | 0.481 |
| **Fruit intake (cups/day)** | 2.2 (5.1) | 1.7 (1.2) | 2.2 (5.3) | 0.502 |
| **Vegetable intake (cups/day)** | 2.2 (4.5) | 1.9 (1.4) | 2.3 (4.6) | 0.657 |

Data are presented as mean (SD) or n (%). BMI, body mass index; CVD, cardiovascular disease.

^a^P-value for the comparison between cases and non-cases: χ^2^ (categorical) or general linear model F-tests (continuous).

^b^Normal weight: <25kg/m^2^, Overweight: 25-29.9; Obese: ≥30kg/m^2^. ^c^Heavy drinking defined as >7 alcoholic drinks/week for females or >14 alcoholic drinks/week for males.

^d^Defined as systolic/diastolic blood pressure ≥130/80mmHg, self-reported physician diagnosis of hypertension, and/or taking blood pressure medication.

^e^Defined as self-reported physician diagnosis myocardial infarction, stroke, and/or congestive heart failure.

^f^Defined as self-reported physician diagnosis of cancer (except skin cancer).

^g^Defined as blood glucose concentration >126mg/dL, self-reported physician diagnosis of diabetes (type 1 or 2), and/or taking diabetes medication.

^h^Defined as low-density lipoprotein cholesterol ≥160 mg/dL, self-reported physician diagnosis of high cholesterol, and/or taking cholesterol medication.

^i^Defined as self-reported physician diagnosis of asthma and/or taking asthma medication.

Bolded values indicate *P* <0.05.
